# LincRNA-p21 Promotes Cellular Senescence by Down-regulating the Wnt/β-catenin Pathway in MPP^+^-treated SH-SY5Y Cells

**DOI:** 10.2174/1386207326666230417103137

**Published:** 2023-08-25

**Authors:** Jianyu Zhu, Lingli Chen

**Affiliations:** 1 Department of Traumatology, The First Affiliated Hospital of Wenzhou Medical University, Wenzhou, 325000, People’s Republic of China;; 2 Department of Neurology, The First Affiliated Hospital of Wenzhou Medical University, Wenzhou, 325000, People’s Republic of China

**Keywords:** Lincrna-p21, senescence, Wnt/β catenin pathway, 1-methyl-4-phenylpyridinium, SH-SY5Y, DAT

## Abstract

**Aim and Objective:**

Long intergenic non-coding RNA-p21 (lincRNA-p21) plays a critical role in various senescence-associated physiological and pathological conditions. We aimed to explore the senescence-associated effects of lincRNA-p21 in 1-methyl-4-phenylpyridinium (MPP^+^) treated neuroblastoma SH-SY5Y cell line as a therapeutic target.

**Materials and Methods:**

The RNA expression levels of lincRNA-p21, p53, p16, and telomere length were examined with reverse transcription-quantitative polymerase chain reaction (RT-qPCR). The Telo TAGGG™ Telomerase PCR ELISA PLUS Kit was used to determine telomerase activity. Cellular viability was evaluated with the 3-(4,5-dimethylthiazol-2-yl)-2,5-diphenyl-tetrazolium bromide (MTT) assay and lactate dehydrogenase (LDH) assay. Western blot was performed to analyze β-catenin protein expression. Besides, oxidative stress was evaluated by J-aggregate-forming delocalized lipophilic cation, 5,5',6,6'-tetrachloro-1,1',3,3'-tetraethylbenzimidazolocarbocyanine++ + iodide (JC-1) stain, fluorescence spectrophotometry, colorimetric assay, and malondialdehyde (MDA) formation.

**Results:**

This research demonstrated that MPP^+^ caused a distinct increase in the expression of LincRNA-p21 in SH-SY5Y cells. MPP^+^ induced cellular senescence with decreasing cellular proliferation and viability, increasing expression levels of senescence-associated makers such as genes p53 and p16, accompanied by significantly decreasing telomere length and telomerase activity. At the same time, these effects were abolished by silencing lincRNA-p21 with small interfering RNA (siRNA). On the contrary, β-catenin silencing contributes to reversing anti-senescent effects caused by lincRNA-p21 silencing. Moreover, modifying lincRNA-p21 exerted an anti-senescent influence depending on decreasing oxidant stress.

**Conclusion:**

Our study showed that in the treatment of MPP^+^, lincRNA-p21 might serve a role in the SH-SY5Y cell senescence by modulating the Wnt/β-catenin pathway, as well as increasing oxidant stress. Thus, trying to target lincRNA-p21 may have important therapeutic and practical implications for PD.

## 1 INTRODUCTION

As one of the most prevalent degenerative diseases worldwide, Parkinson’s disease (PD) is characterized by hidden onset, high early misdiagnosis, and high disability rates [1]. However, the exact etiology and underlying mechanisms of PD remain poorly understood [2]. It is generally believed that PD is caused by the degeneration and death of dopaminergic neurons, resulting in the depletion of dopamine (DA) levels in the substantia nigra [1-3]. Previous studies have shown the contributions of oxidative stress, mitochondrial dysfunction, telomeric disorder, and activation of the senescent and apoptotic cascade in the development of PD [4-7]. Data collected from PD patients in the earliest stages of the disease indicate that heightened oxidative stress is a prominent characteristic of the earliest disease phases, occurring before considerable neuron loss [8]. Oxidative stress is characterized by dysregulation of cellular redox activity, in which the creation of reactive oxygen species exceeds their clearance by endogenous antioxidant enzymes and molecular chaperones [9]. Though oxidative stress is not harmful, the buildup of reactive oxygen species (ROS) following cellular redox imbalance can mediate neuronal damage [9, 10]. Moreover, dopaminergic neurons are sensitive to mitochondrial dysfunction due to their high oxidative metabolism [11]. Meanwhile, telomeric disorder plays an important role in PD. Telomeres are formed by combining DNA at the end of chromosomes with specific proteins that repair DNA damage and maintain stability [12]. Due to the “end-replicating problem”, telomeres get shorter, telomerase activity reduces after each cell cycle until they are worn out, and the cell stops dividing [12, 13]. The shortening of telomere length and reduction of telomerase activity are signs of aging and are essential mechanisms leading to senescence [12]. Finally, these disorders lead to the eventual initiation of the senescent and apoptotic cascade, resulting in the loss of dopaminergic neurons [1, 2].

Current treatments focus on improving motor disorders rather than preventing the neurodegeneration of healthy dopaminergic neurons, leading to PD's continuous progression and irreversibility [1, 14]. Consequently, it is necessary to find new targets for PD treatment to reverse dopaminergic neuron damage and provide neuroprotection to healthy dopaminergic neurons.

LincRNA-p21, which is related to cell proliferation, metabolism, and reprogramming, is considered a potential diagnostic marker of various diseases [15]. Studies of lincRNA-p21 have focused mainly on its role in tumor development, and relatively few studies have examined its relationship to neurodegenerative diseases [16, 17]. Recently, a comprehensive analysis of lincRNA expression levels in brain specimens from twenty patients diagnosed with PD and ten healthy controls found that five lincRNAs were substantially differently expressed between the two groups, among which lincRNA-p21 was up-regulated significantly in PD patients [18]. Notably, the level of lincRNA-p21, which is tightly associated with PD, has been found to be up-regulated during the early phase of PD, even before symptoms become apparent [18, 19]. However, the precise involvement of lincRNA-p21 in PD remained unclear. As functional senescence of dopaminergic neurons plays a critical role in PD and lincRNA-p21 can promote cellular senescence, it is logical to assume such a link between lincRNA-p21 and the occurrence and development of PD [1, 15, 19]. Defining the association between lincRNA-p21 and PD, and the possible mechanisms involved is crucial for the clinical prevention and treatment of the disease.

The Wnt/β-catenin pathway, which can be inhibited by lincRNA-p21, participates in several critical physiological and pathological processes of the central nervous system by regulating cell survival, proliferation, and differentiation [15, 20, 21]. Several studies demonstrated that the Wnt/β-catenin pathway is essential for developing aging-related diseases, and path abnormalities are found in many degenerative diseases [22-24]. Remarkably, the Wnt/β-catenin pathway contributes to regulating not only the subventricular region of the substantia nigra, but also the largest dopaminergic terminal effect region in the adult brain and the Wnt-sensitive region around the midbrain quite near the substantia nigra, which contains neural stem cells with dopaminergic potential [24]. Furthermore, it showed that Wnt/β-catenin governs the survival of dopaminergic neuron and neural stem cells by repairing injury and promoting regeneration to improve PD motor and non-motor symptoms [24].

Given the high level of lincRNA-p21 expression in PD, the neuroprotective effects of Wnt/β-catenin, and recent studies that have demonstrated that lincRNA-p21 could inhibit β-catenin expression, we investigated the function and molecular processes behind lincRNA-p21 in the pathogenesis and development of PD. We chose a 1-methyl-4-phenylpyridinium (MPP^+^) treated neuroblastoma SH-SY5Y cell line, which is one of the most classical PD models, as an *in* *vitro* PD model in our study. MPP^+^ is an active metabolite of 1-methyl-4-phenyl1,2,3,6-tetrahydropyridine (MPTP), a neurotoxin commonly employed to create an in vitro PD model in human dopaminergic cell lines such as SH-SY5Y neuroblastoma cells [25, 26]. MPP^+^ is transported into dopaminergic neurons by the dopamine transporter (DAT), resulting in suppression of mitochondrial complex I and adenosine triphosphate (ATP) synthesis and an increase in reactive oxygen species (ROS), which ultimately results in neurodegeneration [2].

## 2 MATERIALS AND METHODS

### 2.1 Cell Culture and Model Establishment

SH-SY5Y were purchased from the American Type Culture Collection (Manassas, VA, USA) and cultured in DMEM (Gibco, Carlsbad, CA, USA) supplemented with 10% FBS (Gibco) at 37°C. As previously described [27], SH-SY5Y cells were incubated with 1 mM 1-methyl-4-phenylpyridinium (MPP^+^) (Sigma, MO, USA) for 24 h as a PD model.

### 2.2 MTT Assay and Lactate Dehydrogenase (LDH) Assay

The viability of the cultured SH-SY5Y cell line was examined by MTT assay and LDH assay. For the MTT assay, MTT in PBS (Gibco) was added to each well of 96-flat-bottom plates for 4 h before SH-SY5Y harvesting. The absorbance of each well at 570 nm was recorded. For LDH assay, an LDH assay kit (Jiancheng Bioengineering Institute, Nanjing, China) was used to raise the detection level of LDH. According to the protocol, cell culture medium was collected and mixed with LDH reaction buffer for 0.5 h at 25°C. The absorbance was measured at 490 nm.

### 2.3 RT-qPCR

RT-qPCR was used to analyze the levels of target genes, including lincRNA-p21, p53, p16, and β-catenin. Briefly, total RNAs were isolated from the SH-SY5Y cell line using TRIzol reagent (Invitrogen, CA, USA) and reverse-transcription into cDNA by the SuperScript cDNA Synthesis Kit (Invitrogen). RT-qPCR was then carried out with SYBR Premix Ex TaqTM (2×) (Takaba, BIO INC. Japan) and a fluorescent quantitative PCR system (Applied Biosystems). Finally, the 2^-∆∆Ct^ method was used to examine all the data.

### 2.4 The Relative Telomere Length Measurement

According to a previously known method, this study quantified the relative telomere length in SH-SY5Y with the qPCR methodology [28].

### 2.5 Relative Telomerase Activity Measurement

For quantitative analyses of telomerase activity, the telomeric repeat amplification protocol (TRAP) assay, in which telomerase reaction product is amplified by polymerase chain reaction (PCR), was performed using the Telo TAGGG PCR ELISA PLUS kit (Roche Molecular Biochemicals, Mannheim, Germany) according to the manufacturer's instructions.

### 2.6 Western Blot

RIPA buffer was used to extract total proteins. Proteins were separated on SDS-PAGE gels and transferred to PVDF membranes. Primary and secondary antibodies were as follows: β-catenin (#8480, Cell Signaling Technology, Danvers, MA, USA), β-actin (#4970, Cell Signaling Technology), Wnt1 (ab 15251, Abcam), and horseradish peroxidase-conjugated anti-rabbit secondary antibodies (#7074, Cell Signaling Technology).

### 2.7 Plasmid Transfection

SiRNA-lincRNA-p21, Ad-Ctrl, Ad-lnc-p21, and siRNA-β-catenin were designed and synthesized. The SH-SY5Y cell line was transfected with siRNAs by Lipofectamine 2000 (Invitrogen).

### 2.8 Mitochondrial Membrane Potential Assay

After washing in PBS, the SH-SY5Y cell line was incubated with JC-1 (Beyotime Technology, Jiangsu, China). The JC-1 fluorescence was then measured with an ELISA plate reader.

### 2.9 Measurement of intracellular ROS

Intracellular ROS of each group were assessed by 2,7-dichlorodihydrofluorescein diacetate (Beyotine Institute of Biotechnology, Nantong, China). A fluorescence spectrophotometer was used to measure the fluorescence intensity of the cells.

### 2.10 SOD activity

SOD activity in the SH-SY5Y cell line was evaluated with the colorimetric assay kit (Abcam, Cambridge, UK). Briefly, proteins were isolated, and SOD activity was measured with the absorbance at 450nm.

### 2.11 Lipid Peroxidation Assay

The production of MDA was measured using an assay kit to evaluate lipid peroxidation. The SH-SY5Y cell line was incubated with MDA lysis buffer, and the supernatant was then added to thiobarbituric acid. At 532 nm, the absorbance of the samples was measured.

### 2.12 Statistical Analysis

The mean and standard deviation were used to present the data. Differences between the two groups were analyzed with a T-test. The statistical significance of differences among more than two groups was determined by one-way analysis of variance (ANOVA) followed by Tukey's post hoc test. SPSS version 21.0 (IBM SPSS, Armonk, NY, USA) was used for all statistical analyses.

## 3 RESULTS

### 3.1 MPP^+^-induced Cellular Senescence and Up-regulated LincRNA-p21 Expression

**Fig. (1):**
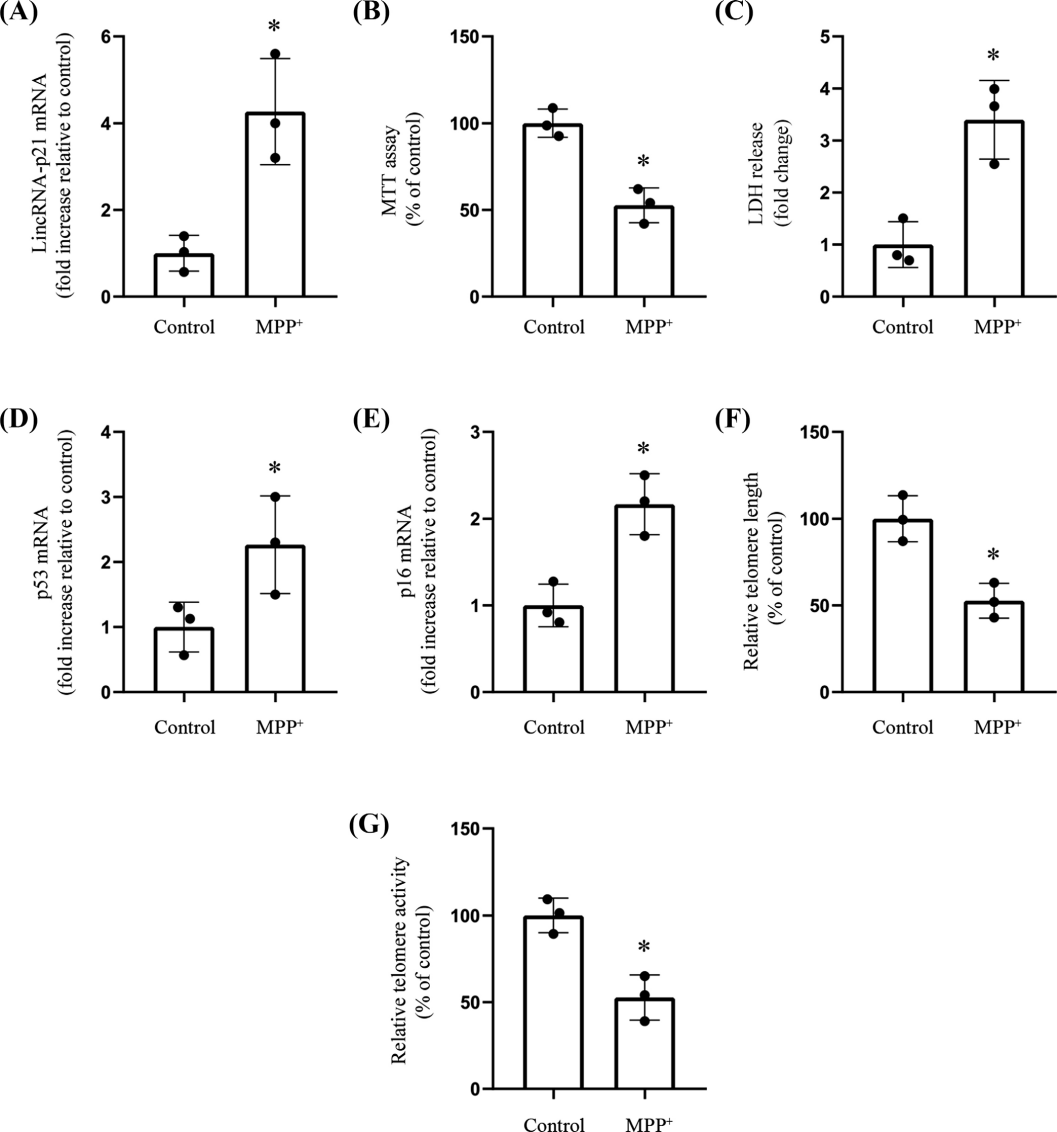
MPP^+^ induced cell senescence and up-regulated the expression level of lincRNA-p21. SH-SY5Y cells were treated with or without MPP^+^. **Notes: (A)** RT-qPCR analyzed lincRNA-p21 mRNA levels (*n*=3; * represents MPP^+^ *vs.* Control). **(B)** MTT assay determined the viability (*n*=3; * represents MPP^+^ *vs.* Control). **(C)** LDH release assessment for cell viability (*n*=3; * represents MPP^+^ *vs.* Control). **(D-F)** RT-qPCR analyses levels of senescence-associated makers such as genes p53 and p16, as well as the telomere length (*n*=3; * represents MPP^+^ *vs.* Control). **(G)** Relative telomerase activity (*n*=3; * represents MPP^+^ *vs.* Control).

The lincRNA-p21 levels in the SH-SY5Y cell line subjected to MPP^+^ were measured and its functions in induced cellular senescence were investigated. Following MPP^+^ treatment, RT-qPCR analysis indicated a considerable increase in lincRNA-p21 expression (Fig. **1A**). Furthermore, after MPP^+^ treatment, the viability of SH-SY5Y decreased significantly (Fig. **1B** and **C**). The MPP^+^-treated group had significantly higher p53 and p16 levels than the controls (Fig. **1D** and **E**). Finally, MPP^+^ treatment reduced telomere length and telomerase activity compared to the control cells (Fig. **1F** and **G**).

### 3.2 MPP^+^-induced Cellular Senescence was Alleviated by Silencing the LincRNA-p21 Expression

**Fig. (2):**
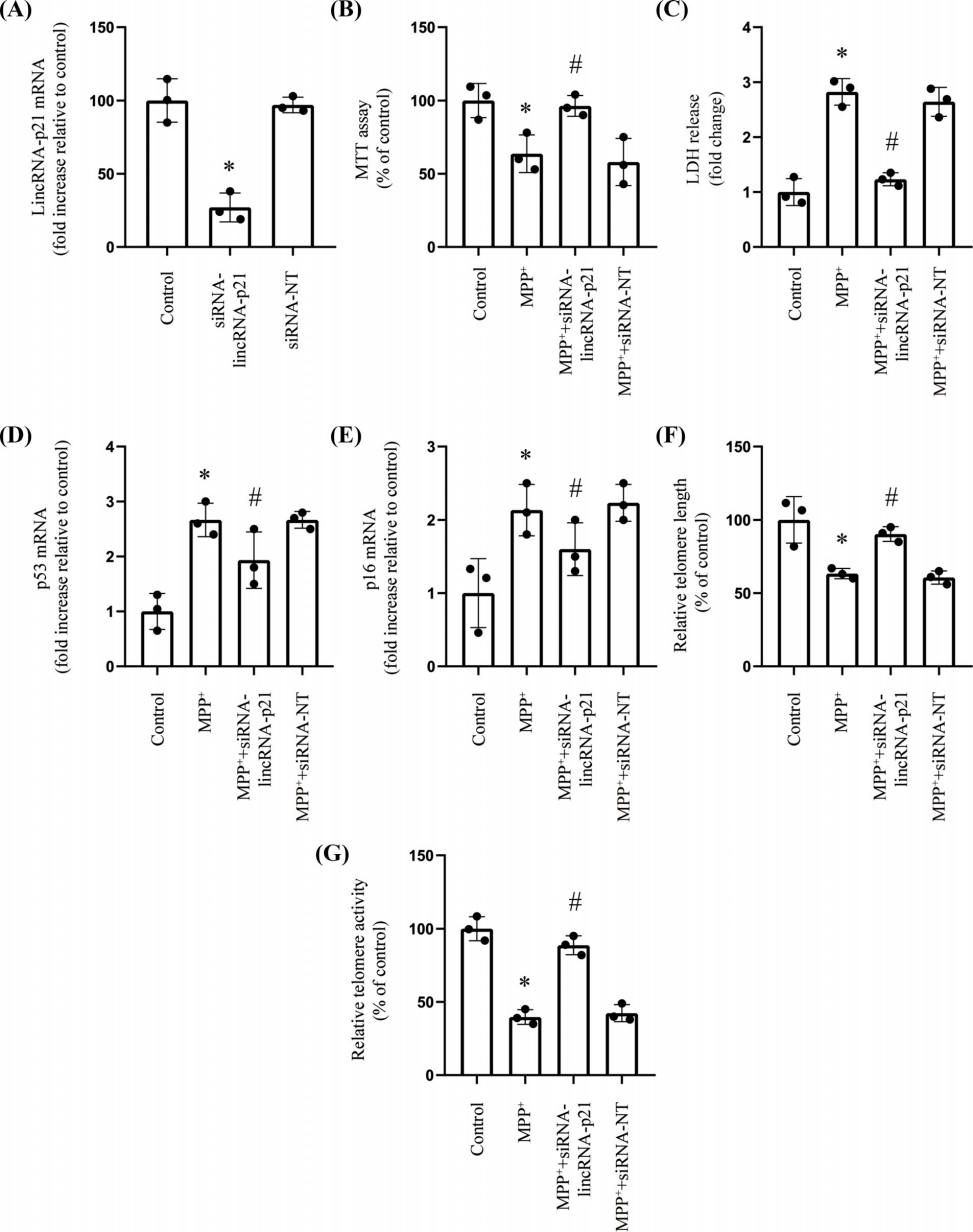
MPP^+^-induced cellular senescence was alleviated by silencing the lincRNA-p21 expression. **Notes: (A)** RT-qPCR was used to analyze the lincRNA-p21 mRNA level (*n*=3; * represents siRNA-lincRNA-p21 *vs.* Control). **(B)** MTT assay determined the viability (*n*=3; * represents MPP^+^ *vs.* Control; ^#^ represents MPP^+^ + siRNA-lincRNA-p21 *vs.* MPP^+^). **(C)** LDH release assessment for cell viability (*n*=3; * represents MPP^+^ *vs.* Control; ^#^ represents MPP^+^ + siRNA-lincRNA-p21 *vs.* MPP^+^). **(D-F)** RT-qPCR analyses levels of senescence-associated makers such as genes p53 and p16, as well as the telomere length (*n*=3; * represents MPP^+^ *vs.* Control; ^#^ represents MPP^+^ + siRNA-lincRNA-p21 *vs.* MPP^+^). **(G)** Relative telomerase activity (*n*=3; * represents MPP^+^ *vs.* Control; ^#^ represents MPP^+^ + siRNA-lincRNA-p21 *vs.* MPP^+^).

The senescence effects of lincRNA-p21 in MPP^+^-induced SH-SY5Y were examined further by siRNA-mediated knockdown of endogenous lincRNA-p21. The RT-qPCR analysis confirmed that the level of lincRNA-p21 in SH-SY5Y cells was reduced following the transfection with a lincRNA-p21-specific siRNA (Fig. **2A**). Cell viability was markedly higher in lincRNA-p21-knockdown cells exposed to MPP^+^ than in control siRNA-NT cells treated with MPP^+^ (Fig. **2B** and **C**). In addition, the knockdown of lincRNA-p21 with siRNA significantly reduced p53 and p16, as well as distinctly increased telomere length and telomerase activity in MPP^+^-treated cells (Fig. **2D**-**G**). By contrast, treatment of SH-SY5Y with a non-specific control siRNA (siRNA-NT) did not alleviate cellular senescence in the presence of MPP^+^.

### 3.3 LincRNA-p21/β-catenin Contributed to the MPP^+^-induced Cell Senescence

**Fig. (3):**
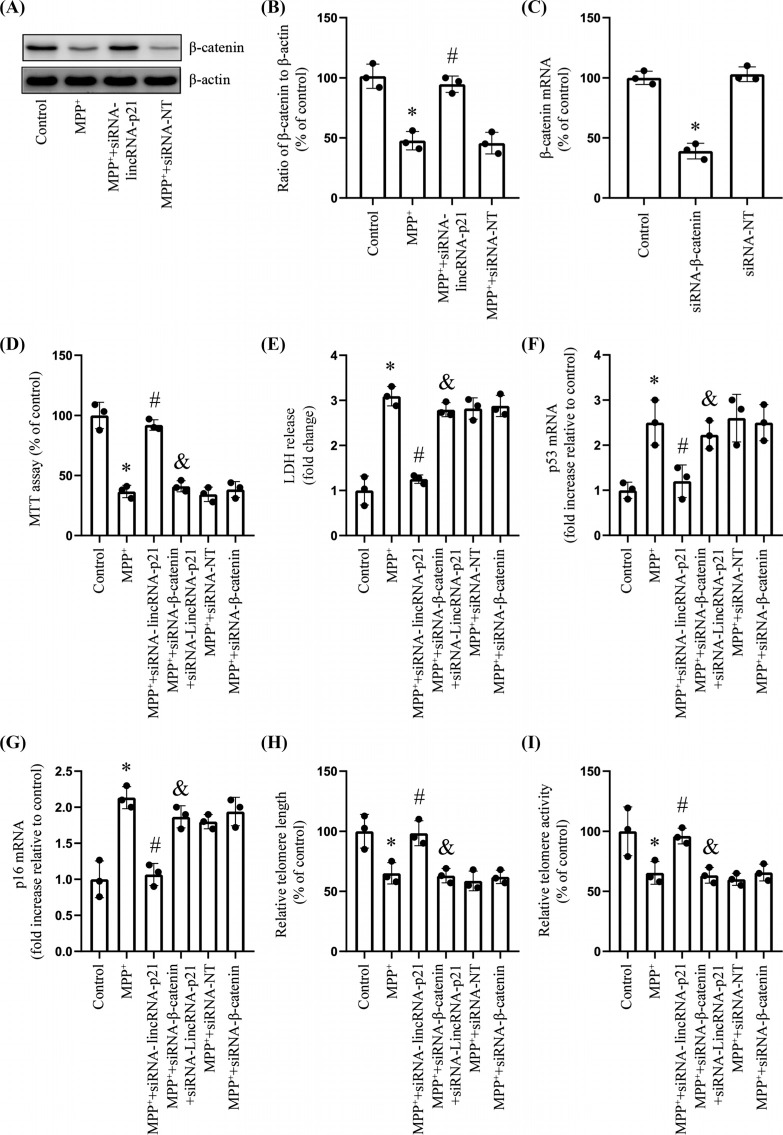
LincRNA-p21/β-catenin signaling pathway related to the cell senescence by MPP^+^-induced cell senescence. **Note: (A and B)** Western blotting analysis the expression level of β-catenin protein (*n*=3; * represents MPP^+^ *vs.* Control; ^#^ represents MPP^+^ + siRNA-lincRNA-p21 *vs.* MPP^+^). **(C)** RT-qPCR analyzed the expression level of β-catenin mRNA (*n*=3; * represents siRNA-β-catenin *vs.* Control). **(D)** MTT assay determined the viability at different times (*n*=3; * represents MPP^+^ *vs.* Control; ^#^ represents MPP^+^ + siRNA-lincRNA-p21 *vs.* MPP^+^; ^&^ represents MPP^+^ + siRNA-lincRNA-p21+ siRNA-β-catenin *vs.* MPP^+^ + siRNA-lincRNA-p21). **(E)** LDH release assessment for cell viability (*n*=3; * represents MPP^+^ *vs.* Control; ^#^ represents MPP^+^ + siRNA-lincRNA-p21 *vs.* MPP^+^; ^&^ represents MPP^+^ + siRNA-lincRNA-p21+ siRNA-β-catenin *vs.* MPP^+^ + siRNA-lincRNA-p21). **(F-H)** RT-qPCR analyses levels of senescence-associated makers such as genes p53 and p16, as well as the telomere length (*n*=3; * represents MPP^+^ *vs.* Control; ^#^ represents MPP^+^ + siRNA-lincRNA-p21 *vs.* MPP^+^; ^&^ represents MPP^+^ + siRNA-lincRNA-p21+ siRNA-β-catenin *vs.* MPP^+^ + siRNA-lincRNA-p21). **(I)** Relative telomerase activity (*n*=3; * represents MPP^+^ *vs.* Control; ^#^ represents MPP^+^ + siRNA-lincRNA-p21 *vs.* MPP^+^; ^&^ represents MPP^+^ + siRNA-lincRNA-p21+ siRNA-β-catenin *vs.* MPP^+^ + siRNA-lincRNA-p21).

Previous studies revealed that lincRNA-p21 down-regulated β-catenin levels [21]. As a result, we investigated the impact of the siRNA-mediated knockdown of lincRNA-p21 on the β-catenin level in the SH-SY5Y cell line. Compared to the negative control group, the MPP^+^-treated group had lower levels of β-catenin protein (Fig. **3A** and **B**). Besides, the knockdown of lincRNA-p21 increased β-catenin expression in the MPP^+^-treated SH-SY5Y cell line (Fig. **3A** and **B**). To examine the relevance of these findings further, we silenced β-catenin using a specific siRNA. As expected, the SH-SY5Y cell line transfected with the siRNA-β-catenin had significantly lower expression levels of the β-catenin mRNA than those transfected with the control siRNA-NT (Fig. **3C**). Furthermore, the aforementioned attenuating effects of lincRNA-p21 knockdown on MPP^+^-induced decreases cell viability, increases in p53 and p16 expression, accompanied by reductions in telomere length and telomerase activity. These effects were significantly reversed by silencing β-catenin (Fig. **3D**-**I**).

### 3.4 The Antioxidant NAC Inhibits the Senescence of SH-SY5Y Caused by MPP^+^

Recent findings have shown that Wnt/β-catenin is related to oxidative stress [29]. As shown in Fig. (**4**), MPP^+^ treatment reduced the mitochondrial transmembrane potential and activity of SOD, as well as increased the expression levels of ROS and MDA in SH-SY5Y cells. However, these effects were diminished by silencing lincRNA-p21. In addition, β-catenin silencing contributed to blocking the anti-senescence effects caused by lincRNA-p21 silencing.

**Fig. (5):**
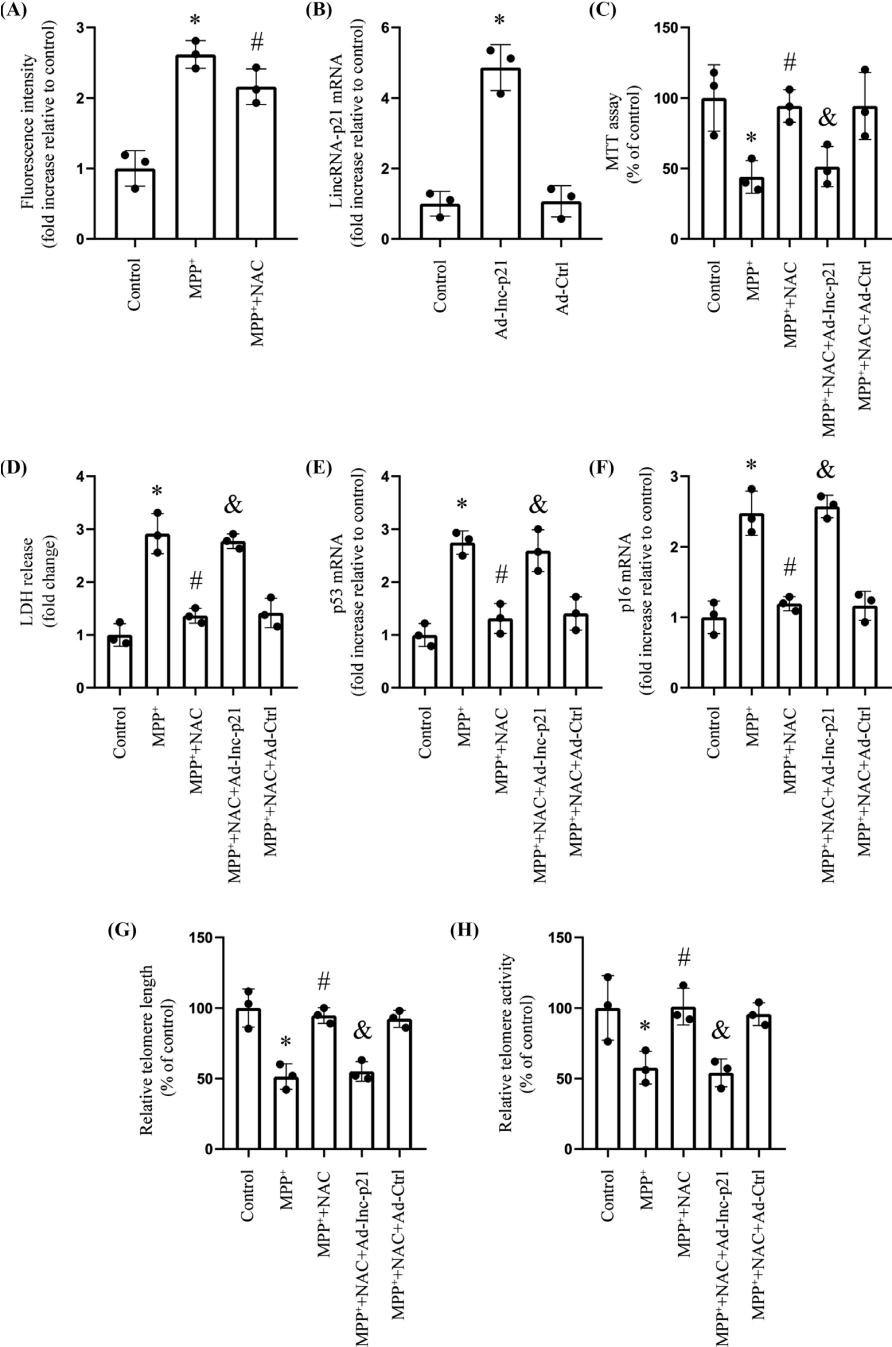
The antioxidant NAC inhibits the SH-SY5Y senescence caused by MPP^+^. **Note: (A)** Fluorescence spectrophotometry measurement of intracellular ROS production (*n*=3; * represents MPP^+^ *vs.* Control; ^#^ represents MPP^+^ + NAC *vs.* MPP^+^). **(B)** We assessed the level of lincRNA-p21 mRNA by RT-qPCR (*n*=3; * represents Ad-lnc-p21 *vs.* Control). **(C)** MTT assay determined the viability (*n*=3; * represents MPP^+^ *vs.* Control; ^#^ represents MPP^+^ + NAC *vs.* MPP^+^; ^&^ represents MPP^+^ + NAC + Ad-lnc-p21 *vs.* MPP^+^ + NAC). (**D**) LDH release assessment for cell viability (*n*=3; * represents MPP^+^ *vs.* Control; ^#^ represents MPP^+^ + NAC *vs.* MPP^+^; ^&^ represents MPP^+^ + NAC + Ad-lnc-p21 *vs.* MPP^+^ + NAC). **(E and G)** RT-qPCR analyses levels of senescence-associated makers such as genes p53 and p16, as well as the telomere length (*n*=3; * represents MPP^+^ *vs.* Control; ^#^ represents MPP^+^ + NAC *vs.* MPP^+^; ^&^ represents MPP^+^ + NAC + Ad-lnc-p21 *vs.* MPP^+^ + NAC). **(H)** Relative telomerase activity (*n*=3; * represents MPP^+^ *vs.* Control; ^#^ represents MPP^+^ + NAC *vs.* MPP^+^; ^&^ represents MPP^+^ + NAC + Ad-lnc-p21 *vs.* MPP^+^ + NAC).

To demonstrate that MPP^+^-induced cellular senescence was specifically caused by lincRNA-p21-mediated oxidative stress, we then attempted to investigate the impact of a well-known powerful antioxidant agent NAC on SH-SY5Y senescence in the presence of MPP^+^. NAC significantly decreased ROS generation in the presence of MPP^+^ (Fig. **5A**). LincRNA-p21 was overexpressed after transfection with Ad-lnc-p21 in the SH-SY5Y cell line (Fig. **5B**). NAC reversed the MPP^+^-induced decreases in cell viability, increases in p53 and p16 expression, as well as reductions in telomere length and telomerase activity of the SH-SY5Y cell line. Nevertheless, transfection of cells with Ad-lnc-p21 abolished these anti-senescence effects (Fig. **5C**–**H**).

## 4 DISCUSSION

Previous research regarding lincRNA-p21 focused mostly on its impact on cancer [15]. However, the function of lincRNA-p21 in the pathogenesis of PD is largely unknown. To the best of our knowledge, our study is one of the few studies to explore lincRNA-p21 in PD. The present study discovered that the neurotoxin MPP^+^ promoted cellular senescence, as demonstrated by decreases in cell viability, increases in p53 and p16 expression, as well as reductions in telomere length and telomerase activity in SH-SY5Y cells.

As a transcription inhibitor, lincRNA-p21 triggers cell damage, senescence, and apoptosis [15]. One study suggested that lincRNA-p21 blocked the JAK2/STAT3 signaling pathway to decrease cell proliferation, thereby inhibiting the progression of head and neck squamous cell carcinoma [16]. Additionally, the lincRNA-p21 expression has been demonstrated to reduce among gastric cancer tissues and cell lines through the β-catenin signaling pathway. Moreover, X-ray irradiation has been proven to increase lincRNA-p21 levels in HCG-27 and SGC7901 gastric cancer cell lines, inhibiting the β-catenin signaling pathway, promoting the aging of cancer cells, and increasing sensitivity to radiotherapy [30]. Consistent with the above findings, we found that MPP^+^ caused a distinct increase in the expression of LincRNA-p21 with decreasing cell viability, increasing expression levels of senescence-associated makers such as genes p53 and p16, accompanied by significantly decreasing telomere length and telomerase activity in SH-SY5Y cells. At the same time, these effects were abolished by silencing lincRNA-p21 with small interfering RNA (siRNA). The above findings suggested that lincRNA-p21 may play crucial functions in regulating neuronal cell senescence in PD and confirmed the results of increasing lincRNA-p21 from human brain specimens [18].

Given the significance of cellular senescence on the development of PD, there is considerable interest in exploring the methods to target senescence for therapeutic purposes [31]. A well-known signaling pathway, Wnt/β-catenin signaling, is highly conserved in genetics and is very similar among different species [22]. Previous research has shown that Wnt/β-catenin signaling is essential for post-injury repair of the nervous system [23, 24]. Notably, lincRNA-p21 can regulate Wnt/β-catenin signaling, thus affecting embryonic development and disease [21]. However, it is unclear whether lincRNA-p21 can affect the occurrence and development of PD by regulating Wnt/β-catenin signaling. In the present research, incubating the SH-SY5Y cell line with MPP^+^ could significantly down-regulated the expression level of β-catenin, while this effect could be abolished by knockdown of lincRNA-p21. By contrast, β-catenin silencing contributes to reversing anti-senescent effects caused by lincRNA-p21 silencing. The limitation is that we did not evaluate the Wnt level with a western blot. Similar to our study, Wei *et al.* reported that activation of the Wnt/β-catenin signaling pathway with exogenous Wnt1 could protect the SH-SY5Y cell line against 6-hydroxydopamine, which is another kind of in vitro PD model [32]. An earlier study has shown that Wnt/β-catenin signaling was necessary for the α7 nicotinic receptor-mediated neuroprotection of dopaminergic neurons in a mouse PD model, and these findings are consistent with our results [33]. Based on previous studies and our observations, we consider that lincRNA-p21/β-catenin contributes to MPP+-induced cell senescence.

Cellular senescence is accompanied by enhanced oxidative stress, such as increased ROS production, increased oxidase activity, and decreased antioxidant enzyme activity [13, 31]. Notably, cellular senescence plays a crucial role in PD [31]. In fact, various hypotheses, such as mitochondrial dysfunction and increased oxidative stress, have been suggested as underlying mechanisms of PD [1, 14, 31]. LincRNAs have been explored and established as critical regulators in the pathogenies of oxidative stress-induced cellular senescence [19]. In particular, lincRNA-p21 is specifically connected to oxidative stress conditions, including DNA destruction and endoplasmic reticulum stress [15, 19]. We found that MPP^+^ induced oxidative stress, as evidenced by reducing the mitochondrial transmembrane potential and SOD activity, as well as increasing the expression levels of ROS and MDA in SH-SY5Y cells. These oxidation effects could be mitigated by silencing lincRNA-p21. Consistent with our findings, Xu *et al.* found that lincRNA-p21 inhibits cell viability and promotes cell apoptosis in PD and Wang *et al.* demonstrated that reducing oxidative stress can improve PD [34, 35]. Of note, in order to further confirm that lincRNA-p21 is related to PD-related oxidative stress, we treated the SH-SY5Y cell line with NAC, a classical antioxidant agent. Our present study showed that NAC could down-regulated senescence in SH-SY5Y cells. Overexpression of lincRNA-p21 eliminated this ameliorative effect, suggesting that down-regulation of lincRNA-p21 could mitigate oxidative stress in PD-associated cellular senescence.

There are some potential agents targeting the PD-involved pathway found, such as hydrogen sulfide, lithium, cannabidiol, and stem-cell-based therapies [29, 36-38].

In summary, we found that in the treatment of MPP^+^, lincRNA-p21 might serve a role in the SH-SY5Y cell senescence by modulating the Wnt/β-catenin pathway, as well as increasing oxidant stress. Based on those results, down-regulating lincRNA-p21 expression to alleviate senescence in neuronal may be a helpful strategy in treating PD.

## CONCLUSION

Our study showed that in the treatment of MPP^+^, lincRNA-p21 might serve a role in the SH-SY5Y cell senescence by modulating the Wnt/β-catenin pathway, as well as increasing oxidant stress. Thus, trying to target lincRNA-p21 may have important therapeutic and practical implications for PD.

## Figures and Tables

**Fig. (4):**
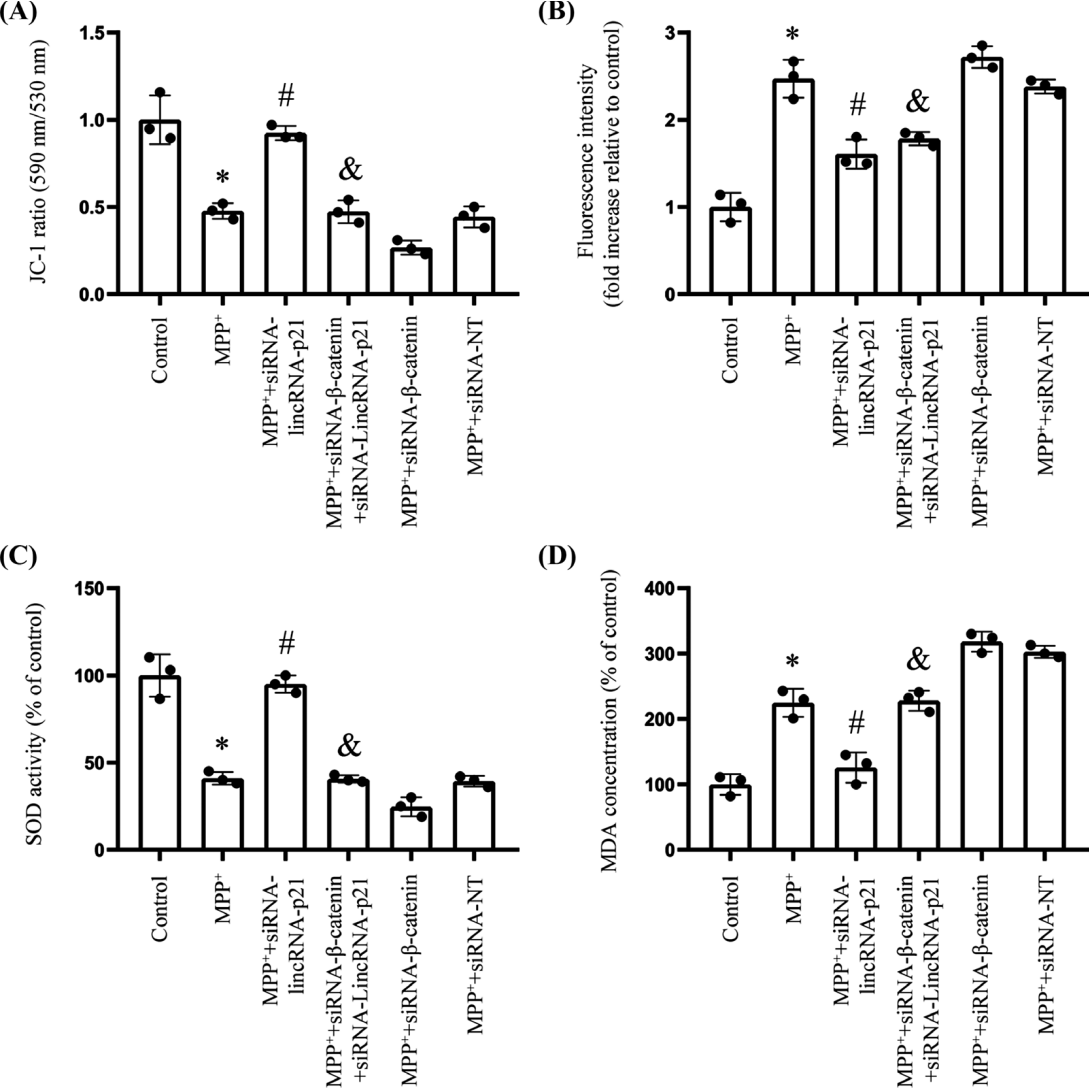
MPP^+^ contributed to the increase of oxidative stress. **Notes: (A)** JC-1 staining as an indicator of the mitochondrial membrane potential (*n*=3; * represents MPP^+^ *vs.* Control; ^#^ represents MPP^+^ + siRNA-lincRNA-p21 *vs.* MPP^+^; ^&^ MPP^+^ + siRNA-lincRNA-p21+ siRNA-β-catenin *vs.* MPP^+^ + siRNA-lincRNA-p21). **(B)** Fluorescence spectrophotometry measurement of intracellular ROS production (*n*=3; * represents MPP^+^ *vs.* Control; ^#^ represents MPP^+^ + siRNA-lincRNA-p21 *vs.* MPP^+^; ^&^ represents MPP^+^ + siRNA-lincRNA-p21+ siRNA-β-catenin *vs.* MPP^+^ + siRNA-lincRNA-p21). **(C)** Colorimetric assay determined SOD activity (*n*=3; * represents MPP^+^ *vs.* Control; ^#^ represents MPP^+^ + siRNA-lincRNA-p21 *vs.* MPP^+^; ^&^ represents MPP^+^ + siRNA-lincRNA-p21+ siRNA-β-catenin *vs.* MPP^+^ + siRNA-lincRNA-p21). **(D)** MDA formation as an indicator of lipid peroxidation (*n*=3; * represents MPP^+^ *vs.* Control; ^#^ represents MPP^+^ + siRNA-lincRNA-p21 *vs.* MPP^+^; ^&^ represents MPP^+^ + siRNA-lincRNA-p21+ siRNA-β-catenin *vs.* MPP^+^ + siRNA-lincRNA-p21).

## Data Availability

Not applicable.

## LIST OF ABBREVIATIONS

PD: Parkinson’s Disease
ROS: Reactive Oxygen Species
DAT: Dopamine Transporter
ATP: Adenosine Triphosphate
LDH: Lactate Dehydrogenase
PCR: Polymerase Chain Reaction
TRAP: Telomeric Repeat Amplification Protocol
